# Impacts of Rootstocks on the Quality Attributes of Tomato Fruit ( *Solanum lycopersicum* ) During Cold Storage

**DOI:** 10.1002/fsn3.70187

**Published:** 2025-04-18

**Authors:** Andaç Kutay Saka, Aslıhan Çilingir Tütüncü, Umut Ateş, Mehtap Özbakır Özer, Harun Özer

**Affiliations:** ^1^ Department of Horticulture, Faculty of Agriculture Ordu University Ordu Türkiye; ^2^ Department of Horticulture, Faculty of Agriculture Ondokuz Mayıs University Samsun Türkiye; ^3^ Department of Horticulture, Faculty of Agriculture Sakarya University of Applied Sciences Sakarya Türkiye; ^4^ Department of Plant and Animal Production, Samsun Vocational School Ondokuz Mayıs University Samsun Türkiye

**Keywords:** antioxidant, grafted seedling, total phenolics, vitamin C, weight loss

## Abstract

The main objective of this study was to assess the effects of rootstocks ([ *Solanum lycopersicum* ] cvs. “Kudret” [KD], “Hamarat” [HD], “Pençe” [PD]) on fruit quality traits and phytochemical components of tomato ( *Solanum lycopersicum* cv. “Depar F1”) during cold storage. Tomato fruit were stored at 8° ± 0.5°C and 90% ± 5% RH for 21 days. Weight loss, respiration rate, soluble solids, titratable acidity, vitamin C, and bioactive compounds (total phenolics, total flavonoids, and antioxidant activity) were determined. At the end of storage, the highest weight loss (%0.18) was determined in tomato fruit of PD combination. On the 7th day, significantly lower respiration rate (4.78 mL CO_2_ kg^−1^ h^−1^) was measured in the fruit of the PD combination compared with the KD and HD combinations. The highest juice pH was obtained from the KD combination on the 14th day (5.0), titratable acidity was obtained from the KD combination on the 21st day, soluble solids content was obtained from the HD combination on the 21st day (%4.40) and the highest vitamin C values were obtained from the tomato fruit harvested from the HD combination on the 14th day (22.9 mg 100 g^−1^). Bioactive compounds decreased in tomato fruit grafted with three different rootstocks on the 21st day compared to the harvest period. In terms of weight loss, the “Kudret” rootstock with the least loss (% 0.06) came to the fore. “Pençe” rootstock stood out in terms of total flavonoid content, “Hamarat” and “Kudret” rootstocks stood out with high antioxidant capacity (DPPH) and “Hamarat” rootstock stood out with high total antioxidant power (FRAP) contents (2.61, 11.94 mmol TE kg^−1^, respectively).

## Graphical abstract

The main objective of this study was to assess the effects of rootstocks on fruit quality traits and phytochemical components of tomato during cold storage. This study has the potential to provide a new perspective for future breeding studies, rootstock–scion combination research, and storage studies by clarifying the effects of rootstock differences on the storage process.

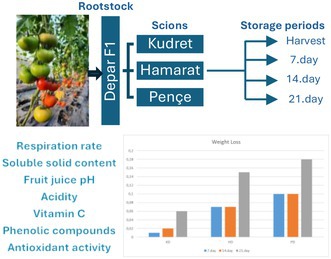

## 1 Introduction

The most produced and consumed vegetable is tomato ( *Solanum lycopersicum* L.) worldwide. It is widely used as a dietary product because it is rich in carbohydrates, fiber, organic acids, amino acids, pigments, vitamins A, C, and E, minerals, and phenolic compounds (Sönmez and Ellialtıoğlu 2014; Khalil et al. 2022; Lee et al. 2023). Total tomato production was 186.1 million tons in 4.9 million hectares of land of the world in 2023 (FAO 2024).

The use of grafted seedlings stands out as an alternative method to increase tolerance to abiotic and biotic stress conditions in vegetable production. Starting production with grafted vegetable seedlings is promising compared to traditional cultivation methods. The use of grafted seedlings contributes to earliness and increased yield by making plants resistant to diseases and pests (Yetişir et al. 2003; Şen and Özenç 2017). While grafted plants show a stronger development, the use of disease and pest resistant rootstocks can also make a significant contribution to reducing the use of pesticides. In addition, the fact that grafted plants have a strong root structure increases the resistance of the plants to soil‐based problems and emphasizes productivity (Tüzel et al. 2005; Ece and Çimen 2013; Tütüncü et al. 2023; Fullana et al. 2023). Today, commercial vegetable grafts are mostly performed on pepper, tomato, watermelon, eggplant, and melon species. It is also considered a quick alternative to laborious breeding processes for developing fruit and vegetable cultivars that are more resistant to environmental stress (Pramanik et al. 2023).

Tomato fruit have a perishable structure, so significant losses occur after harvest (Varela et al. 2003). These losses can vary by 25%–42% depending on factors such as deficiencies in irrigation, feeding, and other cultural processes, pests and diseases, harvest labor, maturity level, and transportation (Pathare and Al‐Dairi 2021; Spricigo et al. 2021; Qasim et al. 2022; Pathare et al. 2023; Walubengo 2023; Roy et al. 2024). There are some studies on the effect of grafting on postharvest losses in tomatoes (Öztürk and Özer 2019; Doltu et al. 2021). However, the number of studies on the effect of rootstock on reducing the severity of postharvest losses of tomato fruit is very limited in the literature.

Therefore, the main study questions were: What is the effect of rootstock on fruit quality characteristics and phytochemical compounds of tomato during cold storage? This research hypothesized that the use of rootstock will have a significant positive effect on maintaining the fruit quality of tomato during cold storage. The objective of the present study was to assess the impact of rootstock on the fruit quality traits and the phytochemical components present in tomatoes during the cold storage period.

## 2 Material and Method

### 2.1 Material

**FIGURE 1:**
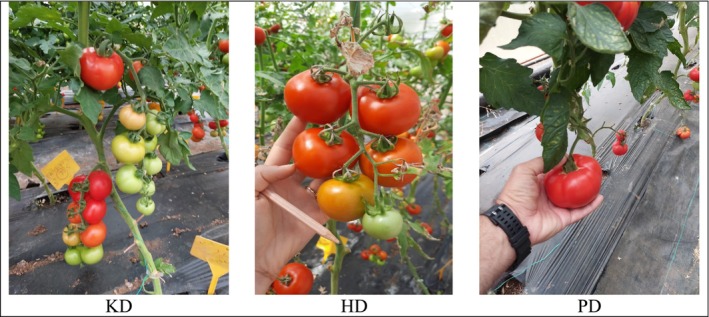
Fruit of tomato plants grafted with different rootstocks used in the study.

The study was carried out in Postharvest Physiology Laboratory of the Department of Horticulture, Ordu University Faculty of Agriculture. Tomato fruit was grown up in the research greenhouses (glass and polycarbonate covered) located in the Research and Application area of the Faculty of Agriculture of Ondokuz Mayıs University between 15 April and 1 July 2022 in Samsun province. After the harvest tomato fruit were transferred to Ordu University Faculty of Agriculture, Department of Horticulture in Ordu province. “Depar F1” tomato cultivar ( *Solanum lycopersicum* ) variety with strong plant growth, high yield, very firm fruits, average fruit weight 180–190 g and long‐life span was used in the study. “Depar F1” was used as a scion in the production of grafted tomato seedlings. “Depar F1” tomato cultivar was grafted on three different rootstocks ( *Solanum lycopersicum* cv. “Kudret,” “Hamarat,” “Pençe”). The rootstocks grafted with “Depar F1” are abbreviated as “Kudret” × “Depar F1”: KD, “Hamarat” × “Depar F1”: HD and “Pençe” × “Depar F1”: PD (Figure 1).

### 2.2 Method

The study involved sowing seeds of tomato rootstocks (“Kudret,” “Hamarat,” “Pençe”) and the scion (“Depar F1”) in 210‐cell trays, each cell measuring 2.6 × 2.6 cm. The trays were filled with peat as the growing medium. Rootstock seeds were planted on April 15, 2022, followed by the scion seeds on April 20, 2022. The seedlings were then transferred to a temperature‐regulated glass greenhouse and placed on growing benches. Throughout the growth phase, irrigation was performed three times daily at 10:00, 14:00, and 16:00, with each session lasting 2 min. Grafting was conducted on May 24, 2022, using the tube grafting technique. After grafting, the seedlings were placed in a specialized intensive care unit within the greenhouse, maintained at 25°C/21°C and 85% relative humidity for 10 days. Following this period, the grafted seedlings were transplanted into their designated positions in the greenhouse.

In greenhouses with a long history of organic vegetable production, raised beds measuring 20 cm in height and 1 m in width were prepared as planting zones. These beds were enriched with 2 kg m^−2^ of burnt animal manure. Drip irrigation lines, spaced 25 cm apart, were installed to accommodate double‐row planting. A soil moisture‐based irrigation system was used throughout the cultivation period. The planting areas were then covered with black polyethylene (PE) mulch. Grafted “Depar F1” tomato seedlings, using KD, HD, and PD rootstocks, were transplanted into the greenhouse at a spacing of 50 cm between rows and plants.

Soil texture analysis for the experimental greenhouse was conducted following the methodology outlined by Kacar and İnal (2008). Temperature (°C) and relative humidity (%) levels in the greenhouse were monitored continuously during the seedling growth phase using a data logger (KT100, Kimo, France), as detailed in Table 1.

**TABLE 1:** Temperature (°C) and relative humidity values in the greenhouse and field.

	Greenhouse	
	Temperature (°C)	Humidity (%)
Lowest	16.1	45.6
Highest	47.3	89.2
Average	28.7	64.4

No supplementary organic fertilizers were applied after planting. The organically cultivated tomato plants were nourished using a custom organic nutrient solution derived from compost water. This solution, with an electrical conductivity (EC) of 1.4 dS m^−1^, was applied at a rate of 500 mL per plant every 15 days. The compost used to produce this nutrient solution was prepared in a composting facility using tomato plant residues, following the pile method described by Inckel et al. (2005). The primary material for the compost was tomato stem waste, cut into 25 cm segments. A 10 cm layer of burnt animal manure was added on top of the tomato waste, followed by a 2 cm layer of garden soil. This layering process was repeated three times, resulting in a compost pile measuring 1.6 × 0.9 × 1 m. The pile was then moistened and covered with a lid. The composting facility was designed with ventilation, allowing consistent monitoring and control of the pile's moisture levels. The compost water, prepared from materials processed in December 2021, was ready for use after 3 months. The nutrient solution was analyzed for pH, EC, nitrogen, phosphorus, and potassium content, following the methods outlined by Kacar and İnal (2008), as presented in Table 2.

**TABLE 2:** Some physical and chemical characteristics of the greenhouse soil and compost fertilizer.

Characteristics	Unit	Greenhouse soil	Compost fertilizer
pH	—	7.30	7.73
EC	dS m^−1^	0.31	3.58
OM	(%)	5.20	—
Nitrogen	%	0.18	0.40
Phosphorus	ppm	12.3	6.90
Potassium	ppm	437	441

Abbreviations: EC, electrical conductivity; OM, organic matter.

Tomato fruit grown organically in the greenhouses of Ondokuz Mayıs University Faculty of Agriculture Research and Application Center were hand‐harvested at the pink maturity stage (fruit with 30%–60% of their surface turned pink or red) according to USDA (1991) standards. After harvest, they were placed into 10 kg plastic crates and transferred on the same day (approximately 2 h later) to the Postharvest Physiology Laboratory of the Department of Horticulture, Ordu University Faculty of Agriculture, using a refrigerated vehicle with conditions of 15°C and 80% humidity. In this study, the fruit from the first four clusters during the harvest period was collected.

All fruit was stored in modified atmosphere packages (MAP). The fruit were packaged into approximately 2.5 kg (20–25 fruit) per package and placed in single layers in 5 kg plastic crates. All the fruit were subjected to precooling with cold air until the fruit temperature reached 10°C.

Subsequently, the openings of the MAP bags were tightly sealed with rubber bands. In the study, StePac (Xtend, Israel) 22 μm LDPE‐based, 5 kg modified atmosphere bags with a CO_2_ permeability of 2203 cc/m^2^.day.atm and a water vapor permeability of 150.0 g/m^2^.day.atm were used at 23°C. The fruit were stored for 21 days in a cold storage room with a temperature of 8°C and RH of 90% ± 5.

During the study, fruit quality analyses and measurements were performed on the 7th, 14th, and 21st days of cold storage, in addition to the harvest period (only control on Day 0). For each measurement period, three repetitions were used for each rootstock. A total of 27 crates (3 × 9 = 27 crates for the 7th, 14th, and 21st days) were stored under cold conditions. Each crate contained approximately 20–25 fruit. During the cold storage period, the following measurements and analyses were carried out as stated in the methods:

#### 2.2.1 Weight Loss Rate (%)

At the beginning of cold storage and in each analysis period, the fruit weight of each replicate was determined by weighing it with a scale sensitive to 0.01 g (Radwag, Poland) and substituting the obtained values in the formula below and expressed as a percentage (Özer et al. 2022). Weight loss measurement during cold storage was carried out in the cold storage in each analysis period:

$$\text{Weight loss}\;\left(\%\right)=\frac{\text{Starting weight}—\text{Final weight}}{\text{Starting weight}}\times 100$$

#### 2.2.2 Respiration Rate

Firstly, the weight and volume of the fruit were determined. Then, the fruit was stored in a 2 L gas‐tight environment (room conditions) for 1 h. The amount of CO_2_ released by the fruit to the external environment was measured with a digital carbon dioxide analyzer (Vernier, USA) and the values obtained were expressed as mL CO_2_ kg^−1^ h^−1^ based on the weight and volume of the fruit placed in the jars (Öztürk et al. 2014).

#### 2.2.3 pH

After the tomato fruit was passed through a hand blender and the juice was filtered during the storage period, analyses were performed on the juice obtained using a pH meter (WTW 3110 Set 2 pH Meter, Germany) (Karaçalı 2009).

#### 2.2.4 Total Soluble Solids (TSS)

For every replication of the rootstock experiment, 10 fruit were selected, and slices from these fruit were blended using an electric mixer. The resulting mixture was then filtered through cheesecloth to extract the juice. Once an adequate sample of the fruit juice was obtained, measurements were taken using a digital refractometer (PAL‐1, McCormick Fruit Tech., Yakima, USA). The readings were recorded and expressed as a percentage (%), following the methodology described by Özer et al. (2022).

#### 2.2.5 Titratable Acidity (TA)

To measure the titratable acidity (TA), 10 mL of fruit juice was mixed with an equal volume of distilled water. The solution was then titrated using 0.1 N sodium hydroxide (NaOH) until the pH reached 8.1. The volume of NaOH used in the titration was converted to citric acid concentration, expressed as g of citric acid per 100 mL of juice (g citric acid 100 mL^−1^), as outlined by Özer et al. (2022).

#### 2.2.6 Vitamin C

Vitamin C content was analyzed using a Reflectoquant Plus 10 device (Merck RQflexplus 10, Turkey), following the protocol described by Uğur et al. (2022). For the analysis, 0.5 mL of the extracted juice was mixed with 0.5% oxalic acid and diluted to 5 mL. An ascorbic acid test kit (Catalog no: 116981, Merck, Germany) was immersed in the solution for 2 s, allowed to oxidize for 8 s, and then inserted into the Reflectoquant device. Readings were taken after 15 s, and the results were reported as milligrams of vitamin C per 100 g of sample (mg 100 g^−1^).

#### 2.2.7 Total Phenolic Compounds

Total phenolic content was determined using the Folin–Ciocalteu method, as described by Peksen et al. (2023). A 400 μL aliquot of fresh fruit extract was combined with 4.2 mL of distilled water, followed by the addition of 100 μL of Folin–Ciocalteu reagent and 2% sodium carbonate (Na_2_CO_3_). The mixture was incubated for 2 h, after which the absorbance of the blue‐colored solution was measured at 760 nm using a spectrophotometer. Results were calculated as gallic acid equivalents and expressed as g per kilogram (g GAE kg^−1^).

#### 2.2.8 Total Flavonoids

Total flavonoid content was assessed according to the method of Öztürk and Özer (2019). A 1 mL aliquot of diluted extract was mixed with distilled water to reach a total volume of 5 mL. Then, 0.3 mL of 5% sodium nitrite (NaNO_2_) was added, followed by a 5 min incubation. Next, 10% aluminum chloride (AlCl_3_) was introduced, and the mixture was left for 6 min. Finally, 1 M sodium hydroxide (NaOH) was added, and the volume was adjusted to 10 mL with distilled water. Absorbance was measured at 510 nm, and the results were expressed as quercetin equivalents (g QE kg^−1^).

#### 2.2.9 DPPH Antioxidant Activity (2,2‐Diphenyl‐1‐Picrylhydrazyl)

The DPPH free radical scavenging activity of the tomato fruit extract was evaluated using a modified version of the method by Blois (1958), as referenced in Öztürk et al. (2016). A DPPH solution served as the free radical source. Stock solutions were prepared at varying concentrations, and 0.5 mL of each was mixed with 0.1 mM DPPH in ethanol. The total volume was adjusted to 3 mL, and the mixture was stirred and left at room temperature for 30 min. Absorbance was measured at 517 nm, and the results were reported as millimoles of Trolox equivalents per kilogram (mmol TE kg^−1^).

#### 2.2.10 FRAP Test [Ferric Reducing Antioxidant Power]

For the ferric reducing antioxidant power (FRAP) assay, 120 μL of extract was combined with 0.2 M phosphate buffer (pH 6.6) to reach a volume of 1.25 mL. Then, 1.25 mL of 1% potassium ferricyanide (K_3_Fe(CN)_6_) was added, and the solution was incubated at 50°C. After incubation, 1.25 mL of 10% trichloroacetic acid (TCA) and 0.25 mL of 0.1% iron chloride (FeCl_3_) were added. Absorbance was measured at 700 nm using a UV–vis spectrometer, and the results were expressed as millimoles of Trolox equivalents per kilogram (mmol TE kg^−1^), following the methods of Benzie and Strain (1996) and Peksen et al. (2023).

#### 2.2.11 Statistical Analysis

The experiment was designed as a Randomized Complete Block Design with split‐plot arrangements and three replications. Each rootstock group included 30 plants, with 10 plants per plot. Data were analyzed using SPSS (version 15.0, New York, USA) through analysis of variance (ANOVA). Mean comparisons were performed using Duncan's multiple range test at a significance level of *p* < 0.05.

## 3 Results and Discussion

### 3.1 Weight Loss and Respiration Rate

The effects of “Depar F1” tomato cultivar grafted on different rootstocks on quality characteristics during cold storage were determined (*p* > 0.05). When the results were analyzed, it was determined that rootstocks had no significant effect on weight loss on the 7th day of storage. However, significant differences were observed between PD and HD on the 14th day. On the 21st day, the weight loss of the fruit belonging to the combination of HD and PD was found to be higher at a similar level with the fruit belonging to KD. In contrast to weight loss, there was no significant difference between the fruit of different rootstocks in respiration measurements on Days 0, 14 and 21. On the other hand, on the 7th day when statistically significant differences were observed, the highest respiration value was determined in KD fruit while the lowest value was determined in PD fruit. The respiration rate of HD cultivar was similar to the other two cultivars (Table 3).

**TABLE 3:** Effects of rootstocks on weight loss and respiration rate of tomato fruit during cold storage.

Storage period/Rootstocks	Weight loss (%)	Respiration rate (mL CO_2_ kg^−1^ h^−1^)
Harvest		
“Kudret”	0	25.09
“Hamarat”	0	26.10
“Pençe”	0	23.53
7 days		
“Kudret”	0.01	7.03a
“Hamarat”	0.07	6.57ab
“Pençe”	0.10	4.78b
14 days		
“Kudret”	0.02b	8.64
“Hamarat”	0.07ab	7.72
“Pençe”	0.10a	5.68
21 days		
“Kudret”	0.06b	6.40
“Hamarat”	0.15a	6.25
“Pençe”	0.18a	7.30

*Note:* Means in columns, within each storage period, followed by the same letters are not different at *p* ≤ 0.05 according to Duncan's test.

In vegetable production, an important factor besides quality is storage. Weight losses that may occur during storage may lead to undesirable results in terms of fruit quality and product income (Arah et al. 2015). In general, weight loss and respiration are two factors that directly affect each other. With increased respiration rates, higher weight loss can occur in the fruits of grafted plants (Ceglie et al. 2016). Weight loss in fruits increases in proportion to the duration of cold storage due to respiration and transpiration (Kader and Yahia 2011). In previous studies, it was determined that weight loss in tomato fruit increased depending on the duration of cold storage (Haile and Safawo 2018; Öztürk and Özer 2019; Mai and Pathare 2021; Pholsin et al. 2024). Factors known to affect weight loss during cold storage of tomato fruit include storage temperature, type of packaging film, gas pretreatment, degree of maturity, and harvest time (Jung et al. 2019). In addition to these factors, there may be differences in weight losses in fruits obtained from grafted tomato plants and different tomato rootstocks. The results obtained from our study also proved this theory. In addition, grafting has significant effects on plant nutrient uptake and water use efficiency by stimulating internal hormone synthesis (Lee et al. 2010). Accordingly, water losses in grafted plants may be high and therefore weight losses and respiration rates may increase.

It is reported that grafting has significant effects on yield and quality (Aktaş 2020). In this study, it was observed that grafting on different rootstocks can be effective in the storage process. Similarly, Öztürk and Özer (2019) reported that inoculation affected respiration rates. Respiration is a series of oxidation–reduction and organic acid consumption events that directly affect the postharvest lifespan of fruits and vegetables (Chumyam et al. 2017). In cold storage, respiration is suppressed and the respiration rate decreases. In our study, an increase was observed in all rootstocks except on the 14th day compared with the previous storage period, and the lowest respiration rate was determined in all varieties at the end of the 21st day. The reason for this increase may be the use of different rootstocks and excessive consumption of biochemical contents in oxidation and reduction events. Öztürk and Özer (2019) also reported similar fluctuations in the respiration rate of tomato fruits stored in cold storage. However, Özer et al. (2022) reported that the respiration rate decreased in all storage periods (7, 14, 21) in tomato fruit stored in a modified atmosphere package at 8°C.

Looking at other grafting studies, Zhao et al. (2011) stated that respiration increased in the storage of grafted melon fruit. In addition, Ilić et al. (2020) reported that rootstock–scion combinations had a significant effect on respiration rate in stored tomatoes. Doltu et al. (2021) also stated that the hardness levels were similar in the storage of fruit from two different cultivars grafted on a rootstock.

According to the findings of our study, significant differences were found in respiration rate. In a study by Walubengo et al. (2022), it was reported that tomato fruit grafted with different rootstocks experienced different rates of weight loss during cold storage. In our study, the least weight loss was observed in tomato plants grafted with KD rootstocks, while more weight loss was recorded in tomato plants grafted with HD and PD rootstocks.

### 3.2 pH, Titrable Acidity, Total Soluble Solids and Vitamin C

When the pH values of the cultivars are analyzed during the storage period, an increasing curve is observed until the 14th day, and then a decreasing curve is observed. However, it was observed that the difference between the pH values of the stored cultivars in this study was insignificant. Similarly, the titrable acidity (TA) contents of the cultivars were statistically indistinguishable on Days 0, 7, and 14 of storage. However, on Day 21, the TA content of the KD cultivar was significantly higher than that of the other cultivars. TSS content, which was at similar levels on Days 7 and 14 of storage, showed significant differences on Days 0 and 21. When vitamin C values were analyzed, it was observed that the content was generally maintained until the 14th day of storage, and then a decrease was observed. In addition, it was determined that there was a significant difference in vitamin C content between the cultivars during the storage period. For example, the highest content was determined from the fruit of the HD combination on Days 0 and 14, while the highest values were measured in the KD and PD cultivars on Days 7 and 14, respectively (Table 4).

**TABLE 4:** Effects of rootstocks on pH, total soluble solids (TSS), titratable acidity (TA), and vitamin C of tomato fruit during cold storage.

Storage period/Rootstocks	pH	TA (g citric acid 100 mL^−1^)	TSS (%)	Vitamin C (mg 100 g^−1^)
Harvest				
“Kudret”	4.6	0.17	4.2b	11.1c
“Hamara”	4.6	0.16	4.8a	17.0a
“Pençe”	4.6	0.16	4.3b	13.0b
7 days				
“Kudret”	4.7	0.15	4.00a	17.60a
“Hamarat”	4.6	0.14	3.60ab	16.50ab
“Pençe”	4.7	0.14	4.00a	12.60b
14 days				
“Kudret”	5.0a	0.11	3.90	20.30b
“Hamarat”	4.8ab	0.10	3.80	22.90a
“Pençe”	4.9ab	0.12	4.00	21.90ab
21 days				
“Kudret”	4.89	0.18a	4.00b	13.00b
“Hamarat”	4.71	0.13b	4.40a	13.10b
“Pençe”	4.88	0.14b	4.30ab	15.70a

*Note:* Means in columns, within each storage period, followed by the same letters are not different at *p* ≤ 0.05 according to Duncan's test.

Abbreviations: TA, titratable acidity; TSS, total soluble solids.

Vaccination is one of the fastest applications to increase yield and quality (Flores et al. 2010). After grafting, some physical and biochemical changes can be observed along with increased quality. Indeed, Nicoletto et al. (2013) reported that there were differences in the contents of tomato fruit such as pH, TSS, and acidity after grafting. Davis et al. (2008) emphasized that criteria such as pH, acidity, and sugar may vary depending on the rootstock. Rouphael et al. (2010) reported that as a result of vaccination, there are experimental opinions that vary in the amount of ascorbic acid. Turhan et al. (2011) stated that different rootstocks generally have no effect on TA and TSS, but have an effect on vitamin C. According to our study data, it was determined that there were significant differences in vitamin C amounts in the storage of different rootstocks. It was determined that the amount of vitamin C showed an irregular curve during the storage period, but in general, there was an increase until the 14th day of storage.

Similar to our findings, Ilić et al. (2020) stated that the vitamin C content of grafted cultivars increased at a similar level with the progress of storage. Walubengo et al. (2022) also stated that vitamin C and titratable acidity content may vary depending on rootstock and cultivar during storage. Doltu et al. (2021) pointed out that sugar, TA, and vitamin C levels of fruit of different cultivars grafted on the same rootstock may differ.

### 3.3 Total Phenolics, Total Flavonoids, DPPH, and FRAP

When the biochemical properties of “Depar F1” tomato cultivar grafted on different rootstocks were evaluated, the highest total phenolic content, total flavonoids, DPPH, and FRAP activities were obtained from the fruit of HD cultivar on Day 0 (*p* < 0.01). On the 7th day of storage, total phenolic content, total flavonoids, and FRAP values of KD and HD cultivars were statistically different from the other cultivars. In addition, only KD cultivar was significantly higher in terms of DPPH activity on the 7th day of storage. On the 14th day of storage, there was no difference between the total phenol and total flavonoid contents of the cultivars. On the contrary, DPPH and FRAP activities of HD cultivar were significantly higher than the other two cultivars on the same measurement period. On the 21st day of storage, there was no difference in total phenolic content between the cultivars, but total flavonoid content was similarly higher in HD and PD cultivars. On the same period, DPPH and FRAP activities were significantly higher in KD and HD cultivars compared with other cultivars (Table 5).

**TABLE 5:** Effects of rootstocks on total phenolics, total flavonoids, and antioxidant activity (DPPH and FRAP assays) of tomato fruit during cold storage.

Storage period/Rootstocks	Total phenolics (g GAE kg^−1^)	Total flavonoids (g QE kg^−1^)	DPPH (mmol TE kg^−1^)	FRAP (mmol TE kg^−1^)
Harvest				
“Kudret”	9.22b	7.71ab	4.79ab	14.88ab
“Hamarat”	11.92a**	8.80a**	6.47a*	19.71a*
“Pençe”	8.43b	6.79ab	3.76ab	12.19ab
7 days				
“Kudret”	15.67a**	10.54a**	5.92a**	17.60a**
“Hamarat”	12.58a	9.48a	2.12b	16.79a
“Pençe”	8.92b	5.07b	1.35b	9.50b
14 days				
“Kudret”	9.51	6.60	3.84b	12.18b
“Hamarat”	11.99	7.25	6.81a**	16.01a*
“Pençe”	13.03	6.26	4.63b	12.18b
21 days				
“Kudret”	8.53	3.52b	2.61a	9.74a
“Hamarat”	8.32	5.76a**	2.61a**	11.94a**
“Pençe”	8.40	7.13a	1.55b	5.41b

*Note:* Means in columns, within each storage period, followed by the same letters are not different at *p* ≤ 0.05 (*) and *p* ≤ 0.01 (**) according to Duncan's test.

Abbreviations: DPPH = 2,2‐diphenyl‐1‐picrylhydrazyl, FRAP = ferric reducing antioxidant power.

It has been reported that some traits are transferred from the rootstock to the plant via xylem (Lee 1994). It has been reported that various results can be obtained in graft combinations depending on the characteristics of the rootstocks used (King et al. 2010). In this context, differences in the biochemical contents of tomato fruit belonging to different rootstocks used in our study and changes in the performance of these fruit during the storage process were determined. In other tomato grafting studies, Milenkovic et al. (2018) reported that total phenols and Marsic et al. (2018) reported that flavonol amounts changed with the effect of grafting. Vrcek et al. (2011) reported that there were significant differences in the biochemical content of tomato cultivars grafted on different rootstocks and that the biochemical content of the fruit of grafted plants decreased in general. Parisi et al. (2023) stated that there were differences in the biochemical content of different cultivars grafted on the same rootstock, but this difference was not significant. In another study, Ilić et al. (2020) stated that the biochemical contents of “Big beef” and “Optima” cultivars grafted to the “Maxifort” cultivar decreased with the effect of vaccination and that the decrease diminished with the progress of storage. With a similar result, Öztürk and Özer (2019) also reported that total phenol, total flavonoid and antioxidant activity decreased with the progress of storage in the study in which grafted tomato fruit were storaged.

## 4 Conclusion

As a result, it was observed that the use of different rootstocks had significant effects on the quality parameters of tomato fruit during cold storage. In general terms, KD rootstock combination stands out in terms of weight loss, and HD rootstock combination stands out in terms of biochemical contents. Tomatoes are mostly selected for consumption according to their qualities such as taste, aroma, appearance, color, and firmness. Therefore, the weight loss, respiration rate, and biochemical content losses that directly affect these quality characteristics of tomatoes should be kept to a minimum level during storage. As a result of this study in which the cold storage potentials of different rootstocks were tested, the effects on the quality characteristics of different rootstocks during storage and the responses of rootstocks to these effects were revealed. Thus, the effects on the quality characteristics of the rootstocks to be selected during storage will be known in future research.

This study has the potential to provide a new perspective for future breeding studies, rootstock–scion combination research, and storage studies by clarifying the effects of rootstock differences on the storage process. In this context, future studies may provide an important contribution to the determination of strategies targeting quality improvement in tomato production.

## Author Contributions

**Andaç Kutay Saka:** data curation (equal), formal analysis (equal), investigation (equal), visualization (lead), writing – original draft (equal). **Aslıhan Çilingir Tütüncü:** data curation (equal), formal analysis (equal), investigation (equal), writing – original draft (equal). **Umut Ateş:** data curation (equal), formal analysis (equal), investigation (equal), writing – original draft (equal). **Mehtap Özbakır Özer:** data curation (equal), formal analysis (equal), investigation (equal), writing – original draft (equal). **Harun Özer:** conceptualization (lead), data curation (equal), investigation (equal), methodology (lead), supervision (lead), writing – original draft (equal).

## Conflicts of Interest

The authors declare no conflicts of interest.

## Data Availability

The data that support the findings of this study are available on request from the corresponding author.
